# Serum Steroid Profiling by Liquid Chromatography–Tandem Mass Spectrometry for the Rapid Confirmation and Early Treatment of Congenital Adrenal Hyperplasia: A Neonatal Case Report

**DOI:** 10.3390/metabo9120284

**Published:** 2019-11-21

**Authors:** Ilaria Cicalini, Stefano Tumini, Paola Irma Guidone, Damiana Pieragostino, Mirco Zucchelli, Sara Franchi, Gabriele Lisi, Pierluigi Lelli Chiesa, Liborio Stuppia, Vincenzo De Laurenzi, Claudia Rossi

**Affiliations:** 1 Center for Advanced Studies and Technology (CAST), University “G. d’Annunzio” of Chieti-Pescara, 66100 Chieti, Italy; ilaria.cicalini@unich.it (I.C.); damiana.pieragostino@unich.it (D.P.); m.zucchelli@unich.it (M.Z.); sara.franchi2@virgilio.it (S.F.); stuppia@unich.it (L.S.); delaurenzi@unich.it (V.D.L.); 2 Department of Medicine and Aging Science, “G. d’Annunzio” University of Chieti-Pescara, 66013 Chieti, Italy; gabriele.lisi@unich.it (G.L.); pierluigi.lelli@unich.it (P.L.C.); 3 Department of Pediatrics, “G. d’Annunzio” University, 66100 Chieti, Italy; stefano.tumini@gmail.com; 4 Department of Pediatrics, “Ospedale della Murgia—Fabio Perinei ”, 70022 Altamura, Italy; pauline.86@hotmail.it; 5 Department of Medical, Oral and Biotechnological Sciences, University “G. d’Annunzio” of Chieti-Pescara, 66100 Chieti, Italy; 6 Department of Psychological, Healthand Territory Sciences, School of Medicine and Health Sciences, “G. d’Annunzio” University, 66100 Chieti, Italy; 7 Department of Pediatric Surgery, Pescara Hospital, 65124 Pescara, Italy

**Keywords:** congenital adrenal hyperplasia, steroid profiling, mass spectrometry, metabolomics, LC–MS/MS

## Abstract

Congenital adrenal hyperplasia (CAH) describes a group of autosomal recessive disorders of steroid biosynthesis, in 95% of cases due to 21-hydroxylase deficiency. The resulting hormonal imbalances lead to increased 17-hydroxyprogesterone and androgens levels, at the expense of decreased concentrations of glucocorticoids and, in some cases, of mineralocorticoids. A variety of clinical presentations accompany a range of severities, which are described as different forms of CAH, and are the result of these hormonal imbalances. The incidence of CAH worldwide is approximately 1 in 15,000 live births, and is population-dependent; thus, its inclusion in neonatal screening tests is widely discussed. Diagnosis of CAH is based on the quantification of 17-hydroxyprogesterone, usually by immunoassay, which has low specificity and high false-positive rates, resulting in a relatively high demand for a second-tier confirmation test. We report a case of a newborn recognized as female at birth, but showing ambiguous genitalia and other CAH clinical features, including hypernatremia, in the first days of life. In addition to the classical assays, liquid chromatography–tandem mass spectrometry was used to determine the serum steroid profile, allowing for the accurate and simultaneous quantification of seven steroids in the same analysis. Such an application immediately revealed an alteration in the levels of specific steroids related to CAH, leading to an early intervention by hormone replacement therapy. Subsequently, the diagnosis of classic CAH due to 21-hydroxylase deficiency was further confirmed by molecular testing.

## 1. Introduction

Congenital adrenal hyperplasia (CAH), the most common adrenal gland disorder in newborns and children, is a group of autosomal recessive disorders characterized by inherited defects in steroid biosynthesis [1]. The cause of about 95% of CAH cases is a 21-hydroxylase deficiency (21-OHD), which is the result of CYP21 gene mutations. In addition, 21-OHD is subclassified in both classical 21-OHD and in non-classical 21-OHD (the late-onset form). Other variants of CAH are caused by a deficiency of 11β-hydroxylase, 17α-hydroxylase, or 3β-hydroxysteroid dehydrogenase [2]. The incidence of CAH worldwide is approximately 1 in 15,000 live births, and is population-dependent [3]. Hormonal imbalances are described as increased levels of 17α-hydroxyprogesterone (17-OHP) and androgens, at the expense of decreased concentrations of glucocorticoids and mineralocorticoids. CAH may include a variety of clinical symptoms, such as poor feeding, failure to thrive, vomiting, dehydration, hyperkalemia, hyponatremia, metabolic acidosis, apathy, virilization, androgen excess, possible salt-wasting crisis as a result of aldosterone deficiency, and incorrect gender assignment [4]. The clinical phenotype strongly depends on the degree of enzymatic impairment, even if the influence of CAH in the development of the biological sex characteristics varies between the sexes. In fact, androgen excess in females with CAH can cause virilization of the external genitalia, also resulting in an altered body, behavior, and sexuality, while androgen excess in male patients is not as profound as in females. In particular, in males, the external genitals are normal, and hyperpigmentation might be observed, but hyperandrogenism causes precocious puberty by accelerating the speed of growth and skeletal maturation, with a subsequent short stature in adulthood [5,6]. Thus, the variety of clinical presentations leads to a range of severities describing different forms of CAH. Neonatal screening for CAH by 17-OHP immunoassay was first introduced in the late 1970s, and in Italy, it is still not mandatory. The 17-OHP immunoassay diagnostic value alone remains questionable, because of the low analytical sensitivity and low diagnostic specificity of such an assay [3,7]. Interestingly, an evaluation of the concentrations of a panel of steroid intermediates in the cholesterol pathway would allow for the detection of the other described enzyme defects. Considering that treatment is possible with an early diagnosis, leading to a substantial reduction in morbidity and mortality, a rapid and simultaneous analysis for the determination of the multiple steroids involved in cortisol (CORT) biosynthesis is strongly required [3,8]. Nowadays, liquid chromatography–tandem mass spectrometry (LC–MS/MS) is a high-throughput analytical technology, often used in routine clinical laboratories and research laboratories, thanks to its ability to quantify a large number of metabolites in a single analysis [9]. Thus, an LC–MS/MS method for the determination of steroid profiling for the rapid confirmation and early treatment of CAH is extremely useful in clinical practice. Here, we describe a case of a baby girl with suspicion of CAH at birth. The application of a steroid profile by LC–MS/MS immediately highlighted the alteration in the levels of some steroids related to 21-OHD, allowing for an early intervention by hormone replacement therapy in the first days of life.

## 2. Case Report

### 2.1. Clinical Presentation

We report the case of a newborn with ambiguous genitalia, who was transferred to the Neonatology Department in Chieti Hospital, Italy. Upon clinical examination, the patient had a small penis/hypertrophic clitoris, two small swellings as bifid scrotum/hypertrophy of the labia majora, a hypospadic urethra with pink mucosa, the absence of palpable testicles as well as vaginal orifice. The child was born from an unrelated marriage, at term, and from spontaneous delivery. The mother had never been exposed to any drugs or hormonal therapy during pregnancy, and had no signs of androgen excess. No family history of infertility, ambiguous genitalia, or unexplained neonatal death was reported. In the first days of life, because of the finding of hypernatremia in the course of physiological weight loss, an infusion of a 5% glucose solution was undertaken, until normalization of the sodium values. Serial electrolyte checks were carried out during the stay, but sodium has been shown to lower the normal limit in a single determination. Upon pelvic and abdominal ultrasound examination, the bladder and uterus were normal-sized for the age; the child reported two masses to the iliac region as ovarian draft, and even in the scrotum/labia, there seemed to be an appreciable gonad for a hemiscrotum (about 6 × 4 mm); no formations referable to testes were adequately assessable in the abdominal regions, and the adrenal lodges were not viewable.

According to the endocrinologist and the pediatric surgeons, in-depth diagnostic investigations were performed as a result of the strong suspicion of classic CAH. The first laboratory tests showed an increase in adrenocorticotropic hormone (ACTH) levels of 250 ng/dL (n.v. 8.0–65.0), and a huge increase in the total testosterone levels of 22.2 ng/mL, 17-OHP levels >20 ng/mL (n.v. <2 ng/mL), renin levels (500 U/mL), and aldosterone levels of 96.4 ng/dL. Moreover, a progressive reduction in cortisol levels from 12.4 to 4.8 μg/dL on the first and eighteenth day of life, respectively, was observed. Given the urgent situation and considering the 17-OHP borderline values, pending the sex determination and the molecular diagnosis, we performed a serum steroid profile by LC–MS/MS. The typical serum steroid pattern, obtained by LC–MS/MS analysis, allowed us to start a replacement treatment with hydrocortisone on the twelfth day of life, at a dose of 10 mg/m^2^/day (1.5 mg/day). Following this, a molecular test confirmed the suspicion of CAH, highlighting a genetic arrangement fully compatible with classic 21-hydroxylase deficiency. After a week of starting hydrocortisone therapy, thanks to the confirmation of a disease normally associated with a loss of salts, we were also able to start a mineralocorticoid replacement therapy (Fludrocortisone, 50 μg/day).

Contextually, a molecular investigation was also performed for sex determination.

### 2.2. Serum Steroid Profiling by LC–MS/MS for Diagnostic Confirmation of Congenital Adrenal Hyperplasia

**Figure 1:**
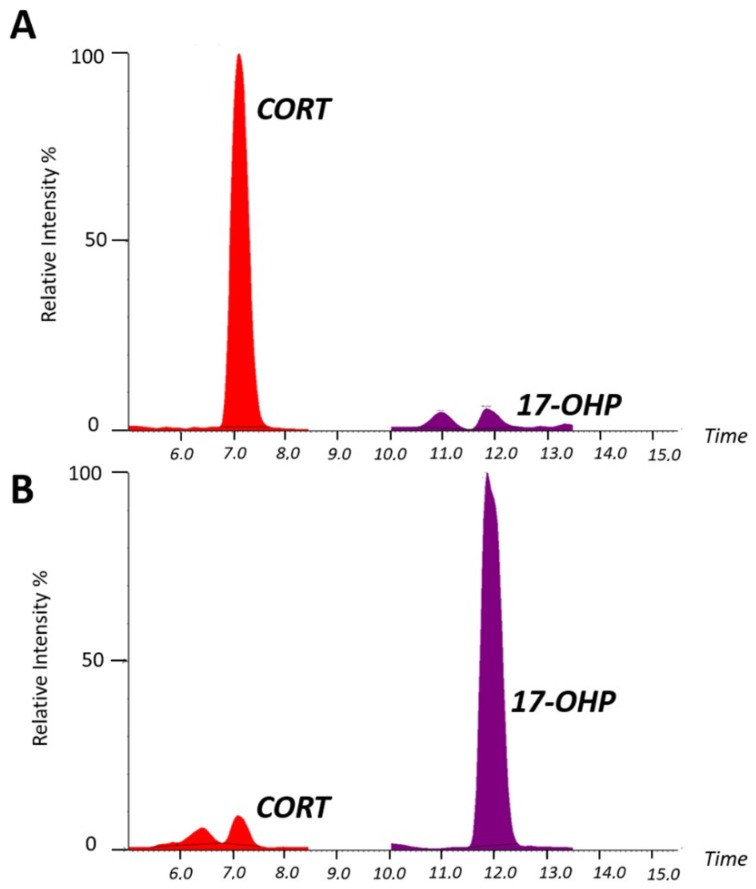
Steroid chromatographic profile. The chromatogram shows the main differences in the serum steroid profile for a normal control sample (Panel (**A**)) and for the sample from the neonatal patient with congenital adrenal hyperplasia (CAH; Panel (**B**)).

First, 100 µL of serum sample was extracted for steroid quantification by LC–MS/MS analysis, using the in vitro diagnostic reagent kit, CHS™ MSMS Steroids Kit (PerkinElmer®, Turku, Finland). Details of the sample preparation and LC–MS/MS analysis are fully reported in the Supplementary Materials (Tables S1 and S2) [10,11]. Briefly, the LC–MS/MS method we performed on the serum sample allowed for the determination and quantification of CORT, corticosterone (CCONE), 11-deoxycortisol (11-DECOL), 4-androstene-3,17-dione (ADIONE), testosterone (TESTO), 17-OHP, and progesterone (PROG) in a single analytical run of 18 mins, using the multiple reaction monitoring (MRM) acquisition mode [10], as previously reported [10,11] and well detailed in Supplementary Materials (Table S2). Figure 1 represents the main differences in the serum steroid profile by LC–MS/MS analysis, between a healthy subject (Panel A) and the neonatal patient with suspicion of CAH (Panel B). The CAH neonatal patient chromatogram (Panel B) is clearly abnormal—the results highlighted a typical steroid pattern, with decreased levels of CORT 3.65 ng/mL (n.v. 10–330 ng/mL), and increased levels of 17-OHP 14.24 ng/mL (n.v. 0.03–2.65 ng/mL). Moreover, the quantified levels of the other monitored steroids for the neonatal patient were CCONE 7.30 ng/mL (n.v. 0.8–18.6 ng/mL), 11-DECOL 1.42 ng/mL (n.v. 0.1–1.56 ng/mL), ADIONE 0.65 ng/mL (n.v. 0.06–2.6 ng/mL), TESTO 0.13 ng/mL (n.v. 0.03–9.7 ng/mL), and PROG 0.61 ng/mL (n.v. 0.07–12.94 ng/mL). In addition, Figure S1 describes the chromatographic separation for each steroid of interest in the related MRM function by the LC–MS/MS analysis of calibration level L7.

#### Analytical Performance of LC–MS/MS Analysis for Serum Steroid Determination

Steroid quantification from the serum patient sample was performed using the calibrators and internal standards provided with the kit. In particular, the calibrators and the quality controls (QCs) consisted of charcoal stripped human serum spiked with seven increasing levels of each steroid analyte. The calibrators were prepared using the same procedure as the patient sample, including the addition of internal standards, as fully described in Supplementary Materials (Tables S1 and S2) [10,11]. The analytical performance, in terms of linearity, showed a coefficient of determination (R^2^ > 0.992) for each steroid, as reported in Figure S2 and Table S3. Moreover, Table S3 also listed the percent coefficient of variation, as calculated by the QuanLynx 4.1 software, for each steroid at low, medium, and high concentration levels (L1, L4, and L7, respectively).

### 2.3. Molecular Testing Confirmation of Congenital Adrenal Hyperplasia

Genomic DNA was isolated from a buccal swab, using a BioRobot EZ1 instrument, according to the manufacturer’s protocol. A molecular analysis of 11 common mutations in the CYP21A2 gene [p.P30L (c.89C>T), I2 splice (c.290-13A/C>G), Del 8 bp E3 (c.329_336del), p.I172N (c.515T>A), cluster E6 (p.I236N, p.V237E, and p.M239K), p.V281L (c.841G>T), p.L307fs (c.920_921insT), p.Q318X (c.952C>T), p.R356W (c.1066C>T), p.P453S (c.1357C>T), and p.R483P (c.1448G>C)] was carried out by a reverse-hybridization test strip-based assay (CAH StripAssay, Vienna Lab Diagnostics GmbH, Vienna, Austria), according to the manufacturer’s protocol [12]. The analysis evidenced the presence of the 515T>A variant in homozygosity (515T>A; 515T>A genotype), causing the substitution of an isoleucine with asparagine in codon 172 of the encoded protein (p. Ile172Asn). In view of the fact that this mutation determines the minimum residual activity of the enzyme (2–11%), the result of the analysis was found to be fully compatible with classic 21-hydroxylase deficiency.

The chromosomal analysis of the patient showed a normal female karyotype (46, XX). A molecular investigation was also performed for sex determination, showing the amplification of the ZFXY marker on both the X chromosomes and the lack of amplification of the SRY gene and the other Y markers. In this way, we excluded the XX male syndrome.

## 3. Discussion

As highlighted in the present case, a newborn with ambiguous genitalia is a clinical emergency, and requires appropriate investigations to be conducted quickly, as a correct diagnosis followed by early treatment is crucial in this inborn error of steroid biosynthesis [13]. It is worth noting that CAH is the most common cause of ambiguous genitalia in newborns, and that 21-OHD represents the cause of about 95% of CAH cases [2,14]. In Italy, as in other national newborn-screening programs, testing for CAH is not mandatory, probably because of the high false-positive rate associated with the traditional immunoassay for 17-OHP determination in dried blood spots [15]. It has been previously reported that the 17-OHP immunoassay is characterized by a low analytical sensitivity and a low diagnostic specificity. In particular, poor immunoassay specificity is a factor in premature newborns or stressed infants, because of the production of high concentrations of delta-5 cross-reacting steroids [3,7,15,16]. In this context, the possibility of a rapid, simple, accurate, and simultaneous quantification of multiple steroids by LC–MS/MS would be useful for reducing the rate of false-positive results in CAH screening, and for improving diagnostic specificity. As the present neonatal case well describes, it is important to note how the application of steroid profiling by LC–MS/MS is crucial for rapid diagnostic confirmation and early intervention for CAH, allowing for improved analytical and diagnostic specificity. Moreover, the high analytical specificity and its potential to quantify several compounds in the same analytical run make metabolic profiling by LC–MS/MS an attractive assay to be used in clinical settings. It is important to emphasize that a CAH positive predicted value by steroid profiling in LC–MS/MS is not only based on an isolated 17-OHP increase, but on the determination of a panel of steroid intermediates. Thus, through steroid profiling by LC–MS/MS, it is possible to discriminate between a higher level of 17-OHP related to CAH, and those related to other, different factors. Furthermore, a steroid profile provides additional clinical information concerning adrenal function, leading to the ability to determine the cause of adrenocortical insufficiency. For example, the inclusion of 21- and 11-DECOL in the panel would highlight the deficient adrenal enzyme, supporting the differential diagnosis of the CAH subtype [1,3]. In addition, considering the over-described CAH variants and the dependence of the clinical phenotype on both the degree of enzymatic impairment and on the sexual genres, steroid profiling by LC–MS/MS would support the specific diagnosis, as well as the correct gender assignment. Finally, the determination of multiple steroids by LC–MS/MS in the diagnostic confirmation of CAH is also useful and informative for the subsequent molecular testing confirmation. In times of molecular genetics, it is worth noting that a snapshot of the metabolic profile may be much more informative and concordant with the clinical phenotype, as not all phenomena in a patient can be understood through the characterization of this genome, as Wudy et al. have already pointed out [7].

In summary, we describe a case of a newborn having ambiguous genitalia and other CAH clinical features, including hypernatremia, in the first days of life. Given the urgent situation, pending the sex determination, a first rapid confirmation of 21-OHD was possible through a biochemical approach by serum steroid profiling in LC–MS/MS. The typical steroid pattern highlighted was crucial for starting early hormone replacement treatment on just the twelfth day of life, and it was also informative for the final confirmation by molecular genetic testing. We believe that the LC–MS/MS method, requiring only a small serum volume and minimal sample preparation, is well suited to become a second-tier test for CAH suspicion. Furthermore, to become a routinely clinical practice, staff highly specialized in MS is strongly recommended. Interestingly, the described approach would avoid expensive and numerous investigations, sparing a patient’s family the anxiety associated with the long wait for diagnostic confirmation.

## Supplementary Materials

The following are available online at https://www.mdpi.com/2218-1989/9/12/284/s1: Table S1: Concentration levels (ng/mL) for calibrators and QC materials of each steroid monitored in the LC–MS/MS method of analysis are summarized. Table S2: MS/MS operating conditions. Multiple reaction monitoring (MRM) functions and settings for detection of steroids are shown. Italics denotes qualifier ion. Table S3: Table shows CV% of calibrator levels at low (L1), medium (L4), and high (L7) concentrations, as well as the R^2^ related to the linear regression for each steroid monitored in the LC–MS/MS method. Figure S1: Multiple reaction monitoring (MRM) chromatogram of calibration level L7, which depicts the quantifying transitions for CORT, CCONE, 11-DECOL, ADIONE, TESTO, 17-OHP, and PROG. Figure S2: The linear regression calculated using the instrumental response (y axis) vs. the concentration in ng/mL (x axis) of the calibrator levels, for the evaluation of the method linearity and for the samples quantification.
