# Supplementary material for: Serum Steroid Profiling by Liquid Chromatography–Tandem Mass Spectrometry for the Rapid Confirmation and Early Treatment of Congenital Adrenal Hyperplasia: A Neonatal Case Report
Source: Metabolites. 2019 Nov 21;9(12):284. doi: 10.3390/metabo9120284 (PMC6950672; doi:10.3390/metabo9120284)
Supplement: Supplementary file 1 [file metabolites-09-00284-s001.pdf]

Supplementary Materials of:

Serum steroid profiling by liquid chromatography–tandem mass spectrometry for rapid confirmation and early treatment of Congenital Adrenal Hyperplasia: a neonatal case report.

Ilaria Cicalini[1,2], Stefano Tumini[3], Paola Irma Guidone[4], Damiana Pieragostino[1,5], Mirco Zucchelli[1,5], Sara Franchi [6], Gabriele Lisi[2,7], Pierluigi Lelli Chiesa[2,7], Liborio Stuppia[1,6], Vincenzo De Laurenzi[1,5] and Claudia Rossi[1,2*]

[1]Center for Advanced Studies and Technology (CAST), University "G. d'Annunzio" of Chieti-Pescara, 66100 Chieti, Italy

[2]Department of Medicine and Aging Science, "G. d'Annunzio" University of Chieti-Pescara, Italy

[3] Department of Pediatrics, "G. d'Annunzio" University, Chieti, Italy

[4]Department of Pediatrics, "Ospedale della Murgia – Fabio Perinei ", Altamura (Bari), Italy

[5] Department of Medical, Oral and Biotechnological Sciences, University ''G. d'Annunzio'' of Chieti-Pescara, Chieti, Italy.

[6]Department of Psychological, Health and Territory Sciences, School of Medicine and Health Sciences, "G. d'Annunzio" University, Chieti, Italy.

[7]Department of Pediatric Surgery, Pescara Hospital, Italy.

* Correspondence: Claudia Rossi, PhD, Department of Medicine and Aging Science, "G. d'Annunzio" University of Chieti-Pescara, Chieti, Italy

e-mail: claudia.rossi@unich.itPhone: +39 0871 541596, Fax: +39 0871 541598

Keywords: Congenital Adrenal Hyperplasia; Steroid profiling; Mass spectrometry; Metabolomics; LC-MS/MS

Materials, samples collection and preparation for serum steroid profile by LC-MS/MS

Cortisol (CORT), corticosterone (CCONE), 11-deoxycortisol (11-DECOL), 4-androstene-3,17-dione (ADIONE), testosterone (TESTO), 17α-hydroxyprogesterone (17-OHP), Progesterone (PROG), and relative internal standard ($^{2}H_{3}$-CORT, $^{2}H_{8}$-CCONE, $^{2}H_{5}$-11-DECOL, $^{2}H_{5}$-ADIONE, $^{2}H_{5}$-TESTO, $^{2}H_{8}$-17-OHP, $^{2}H_{9}$-PROG) were purchased from CHS™ MSMS Steroids Kit, PerkinElmer® (Turku, Finland).

Serum from the patient was collected at Neonatology Department in Chieti Hospital (Italy) using Vacutainer with plain red top cup (Anamedica) for venipuncture (glass tube). Sample was maintained at room temperature (23 ±1 °C) to allow sample coagulation and centrifuged at 4 °C for 15 min at 1400 g. Serum was recovered, avoiding the fluid immediately above the buffy coat layer, and collected in polypropylene tubes.

Each endogenous steroid (1 mg) was dissolved in ethanol and stored at −20 °C. Stock solutions were diluted in methanol/water 50:50 to obtain a final concentration of 0.03 µM (tuning solution). The internal standard mixture (IS mix) from PerkinElmer® kit was reconstituted with 1.25 mL of ACN. Daily Precipitation Solution (DPS) containing Internal Standards was prepared by diluting 1:100 the IS mix with ACN with 0.1% formic acid. Calibrators and quality controls (QCs) were in human serum and were from CHS™ MSMS Steroids Kit, PerkinElmer® (Turku, Finland). Concentration levels (ng/mL) for calibrators and QC materials of each steroid monitored in the LC-MS/MS method of analysis are summarized in Supplementary Table S1.

Table S1

**Analytes**	**Calibration Levels (ng/mL)**							**QC Levels (ng/mL)**		
	L1	***L2***	***L3***	***L4***	***L5***	***L6***	***L7***	***QC1***	***QC2***	***QC3***
CORT	1.51	3.61	8.66	20.8	51.7	129	320	4.32	21.6	160
CCONE	0.29	0.70	1.68	4.03	10.0	24.9	62.0	0.84	4.18	31.0
11-DECOL	0.08	0.20	0.49	1.17	2.91	7.23	18.0	0.24	1.21	9.0
ADIONE	0.08	0.20	0.49	1.17	2.91	7.23	18.0	0.24	1.21	9.0
TESTO	0.03	0.08	0.20	0.47	1.16	2.89	7.20	0.10	0.49	3.60
17-OHP	0.12	0.29	0.70	1.69	4.20	10.5	26.0	0.35	1.75	13.0
PROG	0.12	0.29	0.69	1.72	4.27	10.6	26.5	0.36	1.78	13.2

Table S1: Concentration levels (ng/mL) for calibrators and QC materials of each steroid monitored in the LC-MS/MS method of analysis are summarized.

For steroids quantification by LC-MS/MS analysis, 200 µL of DPS containing IS were added to 100 µL of serum sample, calibrators and QCs, as already reported [1]. Each sample was gently mixed (20 °C, 15 min) in a Thermomixer (Eppendorf®) and then centrifuged (4210 rcf, 20 °C, 30 min). The supernatant (175 µL) was transferred into a new 1.5 mL tube and dried in a SpeedVac for 30 min. The residue was then reconstituted with 125 µL of H_2O/CH_3OH 60:40,

gently mixed in a Thermomixer (20 °C, 15min), briefly centrifuged and supernatant transferred into polypropylene vial (Waters Corporation). The vials were capped, and placed in the system autosampler for the LC-MS/MS analysis.

The LC-MS/MS system consisted of a HPLC Alliance HT 2795 Separations Module coupled to Quattro UltimaPt ESI tandem quadrupole mass spectrometer (Waters Corporation, Milford, MA, USA). The system operated in positive electrospray ionization mode using MassLynx v4.1 software (Waters Corporation, Milford, MA, USA). Autosampler injections of 50 µL were made into sample loop in full mode, with a total run time of 18.00 minutes, injection to injection. For HPLC analysis, the Luna® 3 µm C8, 100 Å, 50 × 3mm column with Security Guard Cartridges, C8, 4.0 × 2.0 mm as guard column (Phenomenex®, USA) was used. The mobile phase comprised a binary solvent system: 98% H2O and 2% MeOH (Solvent A) and 100% MeOH (Solvent B), both containing 0.025% of solvent additive (purchased with the kit). The initial solvent composition was 65% A and 35% B. The mobile phase gradient profile involved three steps: increasing from the initial conditions to 45% B within 4.0 min and then to 65% B within 9.0 min holding for 0.5 min before reaching 100% B until the end of the analysis. The flow rate was 0.35 mL/min and the column was maintained at 45 ℃.

As already reported [2], the mass spectrometer ionization source settings were optimized for maximum precursor ion yields for each steroid. This was achieved by injecting the tuning solution for each individual compound. Functions 1-7 in Table S2 summarize all the parameters refer to the Multiple Reaction Monitoring (MRM) experiments created for each analyte. Two mass transitions were optimized for each analyte, with a single transition being used to monitor the corresponding deuterated internal standards. The first transition was used to quantify the target analyte and the second to qualify the identity of the target compound using a confirmatory ion ratio. The capillary voltage was 3.5 kV, source temperature was 120 °C, desolvation temperature was 400 °C, and the collision cell gas pressure was 3.5 × 10−3 mbar argon. The inter-channel and inter-scan delay times were 0.02 and 0.1 s, respectively. The dwell time was 0.45 s for CORT, 0.30 for CCONE and ADIONE, 0.25 for 11-DECOL, TESTO and 17-OHP, 0.35 for PROG. The same dwell time parameters as the ones of the endogenous compounds were used for the respective internal standards. Data processing and quantification were performed using the QuanLynx 4.1 software (Waters Corporation, Milford, MA, USA) provided with the instrument. Calibration was performed through linear regression with reciprocal fit weighting to ensure maximum accuracy at the lower concentration range.

Table S2

MRM Function	Time Window (min)	Analyte	Transitions (*m*/*z*)	Cone Volts	Coll Energy (eV)
1	5.0–8.5	CORT	363.2 > 120.8	100	20
		CORT	*363.2 > 96.9*	*100*	*20*
		$^2H_3$-CORT	366.2 > 120.8	100	20
2	8.0–10.5	CCONE	347.3 > 120.8	100	18
		CCONE	*347.3 > 96.8*	*100*	*20*
		$^2H_8$-CCONE	355.3 > 124.8	100	18
3	8.0–10.8	11-DECOL	347.3 > 108.8	100	22

		11-DECOL	*347.3 > 96.8*	*100*	*20*
		$^{2}H_5$-11-DECOL	352.3 > 112.8	100	22
4	9.7–12.0	ADIONE	287.2 > 96.9	100	18
		ADIONE	*287.2 > 108.8*	*100*	*20*
		$^{2}H_5$-ADIONE	292.2 > 99.9	100	18
5	10.5–13.0	TESTO	289.2 > 96.8	100	19
		TESTO	*289.2 > 108.8*	*100*	*18*
		$^{2}H_5$-TESTO	294.2 > 99.8	100	19
6	11.2–13.5	17-OHP	331.2 > 96.8	100	21
		17-OHP	*331.2 > 108.9*	*100*	*23*
		$^{2}H_8$- OHP	339.2 > 99.8	100	21
7	13.5–16.0	PROG	315.2 > 96.9	100	18
		PROG	*315.2 > 108.8*	*100*	*19*
		$^{2}H_9$-PROG	324.2 > 99.9	100	18

Table S2: MS/MS operating conditions. Multiple reaction monitoring (MRM) functions and settings for detection of steroids are shown. Italics denotes qualifier ion.

Table S3

	CORT	CCONE	11-DECOL	ADIONE	TESTO	17-OHP
(CV%)L1	-6.9	-4.6	-0.2	0.4	-19.8	-5.2
(CV%)L4	-2.3	-5.8	4.4	1.6	12.1	-7.2
(CV%)L7	-3.2	-3.3	7.9	1.4	3.9	5.9
**R^2**	**1**	**0.999**	**0.998**	**0.999**	**0.997**	**0.992**

Table S3: Table shows CV% of calibrator levels at low (L1), medium (L4) and high (L7) concentration, as well as the R^2 related to the linear regression for each steroid monitored in the LC-MS/MS method.

Figure S1

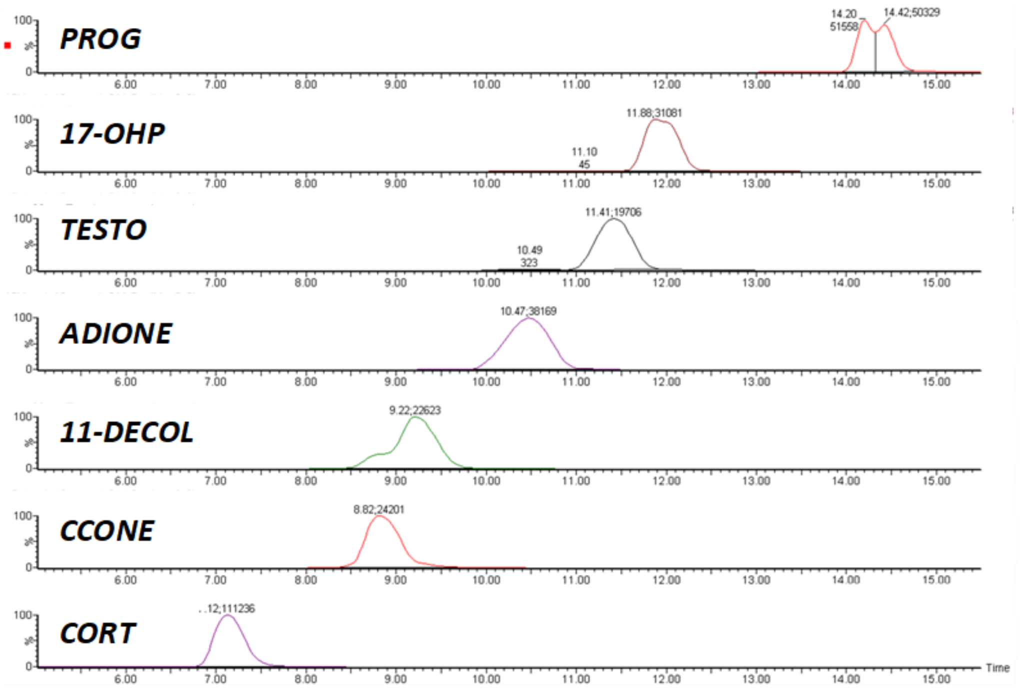

Figure S1: Multiple Reaction Monitoring (MRM) chromatogram of Calibration Level L7 which contains depicting the quantifying transitions for CORT, CCONE, 11-DECOL, ADIONE, TESTO, 17-OHP and PROG.

Figure S2:

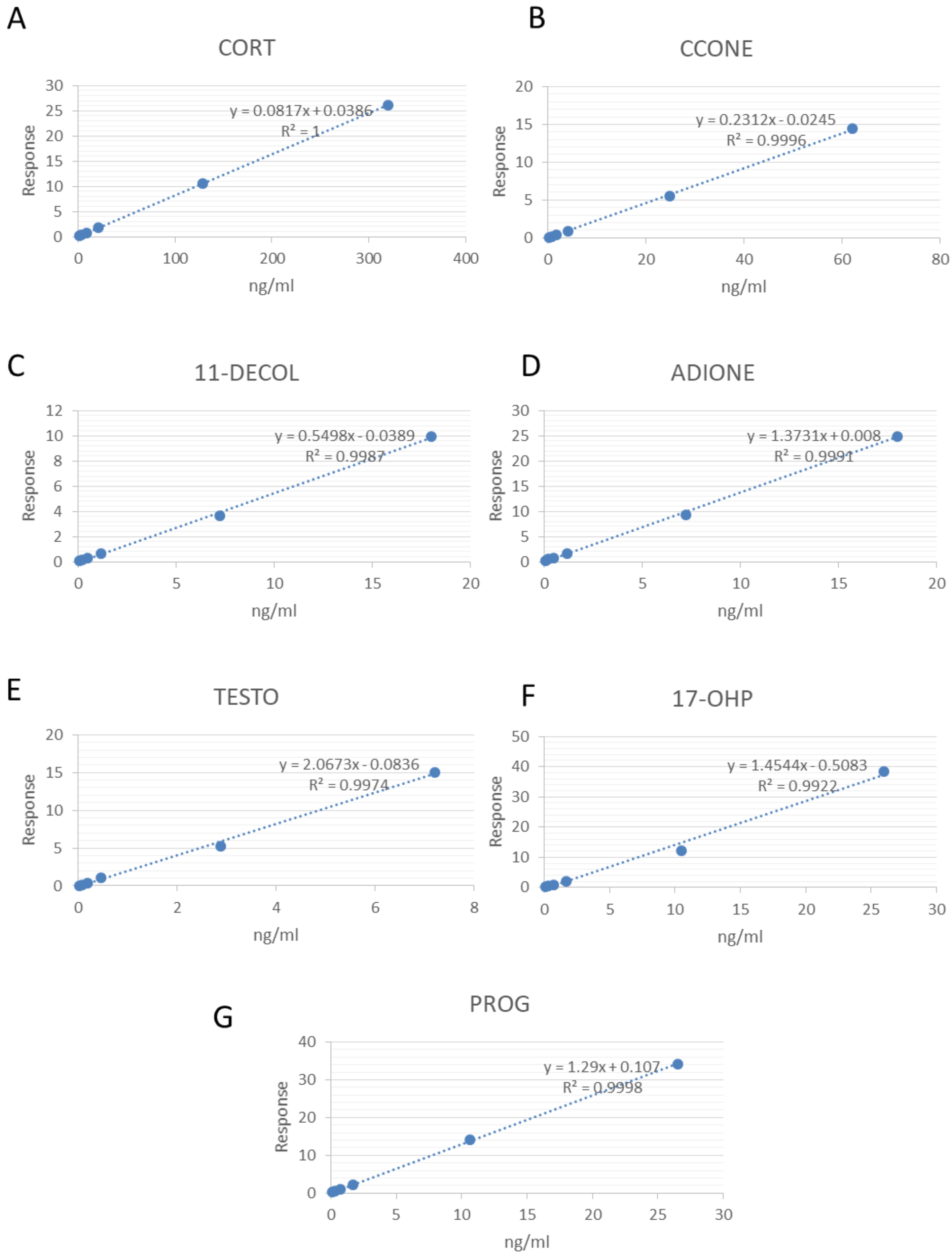

Figure S2: Figure shows the linear regression, calculated by using the instrumental response (y axis) vs the concentration in ng/mL (x axis) of the calibrator levels, for the evaluation of the method linearity and for the samples quantification. (A) linear regression for CORT; (B) linear regression for CCONE, (C) linear regression for 11-DECOL; (D) linear regression for ADIONE; (E) linear regression for TESTO; (F) linear regression for 17-OHP; (G) linear regression for PROG.

References

1. Rossi, C.; Cicalini, I.; Zucchelli, M.; di Ioia, M.; Onofrj, M.; Federici, L.; Del Boccio, P.; Pieragostino, D., Metabolomic Signature in Sera of Multiple Sclerosis Patients during Pregnancy. *International journal of molecular sciences* **2018,** 19, (11).
2. Pieragostino, D.; Agnifili, L.; Cicalini, I.; Calienno, R.; Zucchelli, M.; Mastropasqua, L.; Sacchetta, P.; Del Boccio, P.; Rossi, C., Tear Film Steroid Profiling in Dry Eye Disease by Liquid Chromatography Tandem Mass Spectrometry. *International journal of molecular sciences* **2017,** 18, (7).
